# Nisin-preconditioned mesenchymal stem cells combatting nosocomial *Pseudomonas* infections

**DOI:** 10.1016/j.reth.2024.05.015

**Published:** 2024-06-05

**Authors:** Sara Enayati, Raheleh Halabian, Parvaneh Saffarian, Hossein Aghamollaei, Pardis Saeedi

**Affiliations:** a Department of Biology, Science and Research Branch, Islamic Azad University, Tehran, Iran; b Applied Microbiology Research Center, Biomedicine Technologies Institute, Baqiyatallah University of Medical Sciences Tehran, Iran; c Chemical Injuries Research Center, Systems Biology and Poisonings Institute, Baqiyatallah University of Medical Sciences, Tehran, Iran

**Keywords:** Mesenchymal stem cells (MSCs), Preconditioning, Nisin, Antimicrobial peptide, Wound healing

## Abstract

**Background:**

Nosocomial infections caused by multidrug-resistant *Pseudomonas aeruginosa* are a considerable public health threat, requiring innovative therapeutic approaches.

**Objectives:**

This study explored preconditioning mesenchymal stem cells (MSCs) with the antimicrobial peptide Nisin to enhance their antibacterial properties while maintaining regenerative capacity.

**Methods:**

Human MSCs were preconditioned with varying concentrations of Nisin (0.1–1000 IU/mL) to determine an optimal dose. MSCs preconditioned with Nisin were characterized using microscopy, flow cytometry, gene expression analysis, and functional assays. The effects of preconditioning on the viability, phenotype, differentiation capacity, antimicrobial peptide expression, and antibacterial activity of MSCs against *Pseudomonas aeruginosa* were tested *in vitro*. The therapeutic efficacy was evaluated by topically applying conditioned media from Nisin-preconditioned versus control MSCs to infected wounds in a rat model, assessing bacterial burden, healing, host response, and survival.

**Results:**

An optimal Nisin dose of 500 IU/mL was identified, which increased MSC antibacterial gene expression and secretome activity without compromising viability or stemness. Nisin-preconditioned MSCs showed upregulated expression of LL37 and hepcidin. Conditioned media from Nisin-preconditioned MSCs exhibited about 4-fold more inhibition of *P. aeruginosa* growth compared to non-preconditioned MSCs. In the wound infection model, the secretome of Nisin-preconditioned MSCs suppressed bacterial load, accelerated wound closure, modulated inflammation, and improved survival compared to standard MSC treatments.

**Conclusion:**

This study explores the effect of preconditioning MSCs with the antimicrobial peptide Nisin on enhancing their antibacterial properties while maintaining regenerative capacity. Secreted factors from Nisin-preconditioned MSCs have the potential to attenuate infections and promote healing *in vivo*. The approach holds translational promise for managing antibiotic-resistant infections and warrants further development. Preconditioned MSCs with Nisin may offer innovative, multifaceted therapies for combating nosocomial pathogens and promoting tissue regeneration.

## 1 Introduction

Nosocomial infections associated with multidrug-resistant *Pseudomonas aeruginosa* are a substantial public health crisis. These infections, acquired in healthcare settings, are characterized by antibiotics resistance, leading to higher mortality rates, extended hospital stays, and economic burdens [1]. Hence, inventing more effective therapeutic strategies to address this urgent health concern is imperative.

Regenerative medicine has recently experienced a significant evolution due to the incredible therapeutic potential of mesenchymal stem cells (MSCs). These special cells are remarkably capable of differentiating into various cell types and releasing bioactive molecules. Consequently, they are promising for treating numerous diseases and injuries [[2], [3], [4]]. However, fully harnessing their therapeutic capabilities necessitates innovative strategies to enhance their biological properties.

An innovative approach to improve the effectiveness of MSCs involves using Nisin, a natural antimicrobial peptide. Nisin is well-known for its antibacterial properties and has traditionally been used in the food industry as a natural preservative [5]. However, recent research has discovered its potential for more than just food preservation. It has shown immunomodulatory and antimicrobial effects, leading scientists to explore its intersection with stem cell biology and antibacterial mechanisms [6]. This new application holds promise for enhancing the therapeutic capabilities of MSCs.

In this study, we delve into the world of “Antibacterial Effects of Nisin Preconditioned Mesenchymal Stem Cells.” This exciting new field holds significant implications for regenerative medicine and health as a whole. By examining how Nisin preconditioning affects the behavior and therapeutic potential of MSCs, our goal is to provide valuable insights that can lead to innovative treatment approaches [7]. This research is especially relevant in contexts where addressing bacterial infections and promoting tissue regeneration are crucial priorities.

## 2 Materials and methods

### 2.1 *In vitro* experiments

#### 2.1.1 Human mesenchymal stem cell culture

Bone marrow-derived human MSCs were obtained commercially from Royan Stem Cell Bank (RSCB0178, Tehran, Iran). Stem cells were cultured in Dulbecco's Modified Eagle Medium- High Glucose (DMEM-HG) supplemented with 10% fetal bovine serum (FBS) and 1% penicillin-streptomycin (all from Gibco, USA), incubated at 37 °C, 5% CO2 and 95% humidity. The medium was changed every 3–4 days until cells achieved 70–80% confluence. For passaging, hMSCs were detached, adding 0.25% trypsin-EDTA (Gibco, USA). The cells were seeded in T25 filter-capped culture flasks (SPL, Korea) at a density of 5000 cells/25 cm^2^. Cells between passages 2–5 were used for all following experiments [8].

#### 2.1.2 Nisin preconditioning

Pharmaceutical grade Nisin powder (Sigma Aldrich, USA) was reconstituted in sterile water to prepare a stock solution of 10^6^ IU/mL concentration. The stock was sterilized using a 0.22 μm syringe filter (Millipore, USA). hMSCs were seeded in 96-well plates at a density of 1 × 10^4^ cells per well and allowed to adhere overnight. hMSCs were preconditioned with Nisin concentrations ranging from 0.1 to 1000 IU/mL (equal to 1 μg/mL) prepared by diluting the stock solution in DMEM medium. Untreated HMSCs without Nisin served as control [6]. All confirmatory tests, indicating that the hMSCs had retained their stemness and mesenchymal properties following preconditioning, were conducted [6].

#### 2.1.3 MTT assay

Tracking overnight Nisin preconditioning, cell viability was evaluated via the MTT (3-(4,5-Dimethylthiazol-2-yl)-2,5-Diphenyltetrazolium Bromide) assay. The medium was replaced with 100 μL of 0.5 mg/mL MTT (Sigma-Aldrich, USA) in DMEM and incubated for 4 h at 37 °C. The supernatant was aspirated off the MTT, and 100 μL dimethyl sulfoxide (DMSO, Sigma-Aldrich, USA) was added per well at room temperature for 10 min. Absorbance at 570 nm was measured by a microplate reader (BioTek, USA). Cell viability was calculated as a percentage of control hMSCs. All experimentations were executed in triplicates [9].

#### 2.1.4 Oxidative stress and serum deprivation stress exposure

Cells were seeded at a density of 1 × 10^5^ cells per well of 96-well plates and allowed to adhere overnight. They were then preconditioned with a sublethal dose of Nisin. The protective effects of Nisin preconditioning against oxidative stress were evaluated by exposing hMSCs to 200 μM H_2_O_2_ for 2 h at 37 °C. In contrast, hMSCs were cultured in serum-free DMEM and for 24 h in 2% FBS (serum deprivation). Control cells without Nisin preconditioning were also exposed to H_2_O_2_ and serum deprivation under similar conditions. Ultimately, cell apoptosis was analyzed in preconditioned and controlled hMSCs under both stress conditions by MTT assay and flow cytometry [10].

#### 2.1.5 Antibacterial assay

Initially, human mesenchymal stem cells (hMSCs) were preconditioned with Nisin in a serum and antibiotics-free medium for 24 h. The conditioned medium containing the hMSC secretome was collected and frozen at −20 °C. Bacterial inoculum was prepared by overnight cultures of *P. aeruginosa* (ATCC 10145) in Mueller Hinton broth. Antibacterial activity against *P. aeruginosa* was assessed by a modified micro-broth dilution susceptibility assay, modified well diffusion method, and Real-time PCR assessment of antimicrobial peptides genes expression.

##### 2.1.5.1 Modified micro-broth dilution susceptibility assay

A serial dilution of the Nisin-preconditioned hMSC supernatants was created, and then 100 μL of *P. aeruginosa* fresh culture was added in each well of the 96-well plate. Following overnight incubation at 37 °C in a 5% CO_2,_ bacterial growth in each well was assessed by a microplate reader spectrophotometer (Tecan, Infinite M200 PRO, Switzerland) at 600 nm. Colony counting was also performed by plating serially diluted samples from each well on agar plates to quantify the MIC and MBC values of bacterial growth inhibition more precisely.

##### 2.1.5.2 Modified well diffusion method

The *P. aeruginosa* fresh culture was uniformly plated onto sterile Mueller Hinton agar plates. Immediately, several wells (6 mm in diameter) were created on the medium surface, filling with CMs. After incubation, the diameters of the inhibition zones were measured around each well using measuring calipers or a ruler and compared between groups.

##### 2.1.5.3 Real-time PCR assessment of antimicrobial peptides expression

MSCs were cultured under normal conditions and preconditioned with Nisin (500 IU/mL∼ 0.5 μg/mL) for 24 h. Untreated MSCs were included as the control group. Total RNA was extracted from control and Nisin-preconditioned MSCs using an RNA extraction kit (Pars tous, Iran). cDNA was synthesized by Easy™cDNA synthesis kit (Pars tous, Iran). Briefly, a final volume of 20 μl reactions included 5 μg of each RNA sample, along with 10 μl buffer-mix and 2 μl enzyme-mix. The reaction mixture was incubated at 15–25 °C for primer annealing, 42–45 °C for cDNA synthesis, and 55 °C for denatured RNA-cDNA complex and secondary structures in a thermal cycler, repeated 12 times and finally incubated at 95 °C for 5 min to inactivate the RT enzyme.

Real-time PCR primers were designed for antimicrobial peptides like *LL37*, *hepcidin*, and *gapdh* genes by primer3 online software (http://simgene.com/Primer3) (Table 1). Real-time PCR reactions were performed in a Thermocycler (Qiagen Rotor-Gene Corbett Research, USA). A final volume of 20 μl reactions consisted of 13 μl SYBR Green master mix (2×), 1 μl of each primer, and 1 μl CDNA. The reaction mixture was undergone a 10 min 95 °C, 40 cycles of 30 s 94 °C, 30 s 57–61 °C and 30 s 72 °C. Data were analyzed using the ΔΔCt method to determine relative fold-changes in AMP expression in Nisin-preconditioned vs untreated control hMSCs.

**Table 1:** Primer sequences of antimicrobial peptides used for real-time quantitative PCR (q-PCR) analysis.

Genes	Forward Primer	Reverse Primer	Tm (°C)
*LL-37*	5′-AGGATTGTGACTTCAAGAAGG-3′	5′-GTTTATTTCTCAGAGCCCAGA-3′	59
*hepcidin*	5′-CCCATGTTCCAGAGGCGAAG-3′	5′-CTTGCAGCACATCCCACACT-3′	60
*gapdh*	5′-AGAAGGCTGGGGCTCATTTG-3′	5′-AGGGGCCATCCACAGTCTTC-3′	57

### 2.2 *In vivo* experiments

#### 2.2.1 Animal grouping

In order to investigate the effect of metabolites from preconditioned MSCs in the treatment of a traumatic animal model, 30 adult Sprague-Dawley rats, aged six weeks and weighing between 170 and 180 g, were obtained from the animal care center of Baqiyatallah University of Medical Sciences. The experimental protocol of these studies was approved by the Institutional Animal Care and Use Committee (Approval ID: IR.BMSU.REC.1397.185). Rats were housed under standard conditions at 21 °C with a 12-h light and 2-h dark cycle, with free access to water and standard food. Rats were randomly divided into 5 groups (n = 6 in each group), and were randomly assigned to different treatments. Groups were including; rats with wounds receiving only base ointment as Positive Control (Cont.+), Negative Control-Healthy rats (Cont.-), treated-rats with wounds receiving MSCs’ conditioned medium (MSC-CM), treated-rats with wounds receiving CM from MSCs preconditioned with Nisin (Nisin-MSC-CM), and treated-rats with wounds receiving a antibiotics (Piperacillin/Tazobactam).

#### 2.2.2 Animals wound model

Wounds were made on the backs of rats in 4 groups using a punch biopsy. The resulting wounds were then infected subcutaneously with a pure culture of 1 × 10^7^ *P. aeruginosa* bacteria around the wound area.

#### 2.2.3 Animals treatment with CMs

Rats were treated with a water-soluble base ointment (Samine Soft Cream with 5% urea, Iran) at a 2.5% W/W ratio. The optimal concentration (50 μg) was dissolved in 200 μL of distilled water for both concentrated and non-concentrated culture medium to prepare the relevant ointments. Then, each sample solution was added to the ointment and thoroughly homogenized. Local application on the wounds was performed for 14 days following wound induction.

#### 2.2.4 Hematological analysis

Blood samples were collected in a tube containing EDTA for a complete blood count (CBC) test and the analysis of peripheral blood mononuclear cells to investigate the level of contamination and infection in the animal model.

#### 2.2.5 Wound healing assessment

Samples were collected from the infected wound site and cultured to assess the presence, growth, and bacterial count five days after infection induction. Additionally, wound measurements were taken every other day using a measuring tape for wound size and healing assessment.

For wound site culture, samples were obtained from the wound area using a sterile swab and immersed in 1 mL of PBS. Subsequently, the prepared solution was diluted tenfold and cultured on agar plates, followed by overnight incubation at 37° Celsius. Finally, colony counting was performed.

#### 2.2.6 Animal survival assessment

The survival of rats was evaluated over two weeks. During periodic inspections, the rats' survival status was checked and recorded each day.

### 2.3 Statistical analysis

All experiments were conducted in triplicate with three replicates each, and data were analyzed using SPSS software with one-way ANOVA (analysis of variance) for comparing multiple groups, followed by Tukey's post-test for parametric data analysis. Independent group comparisons were analyzed using the non-parametric Mann-Whitney *U* test. The results were presented as mean ± standard deviation (mean ± SD), and statistical significance was reported for p-values less than 0.05.

## 3 Results

### 3.1 *In vitro* results

#### 3.1.1 Nisin preconditioning of hMSCs

##### 3.1.1.1 dhMSCs viability following Nisin preconditioning

**Fig. 1:**
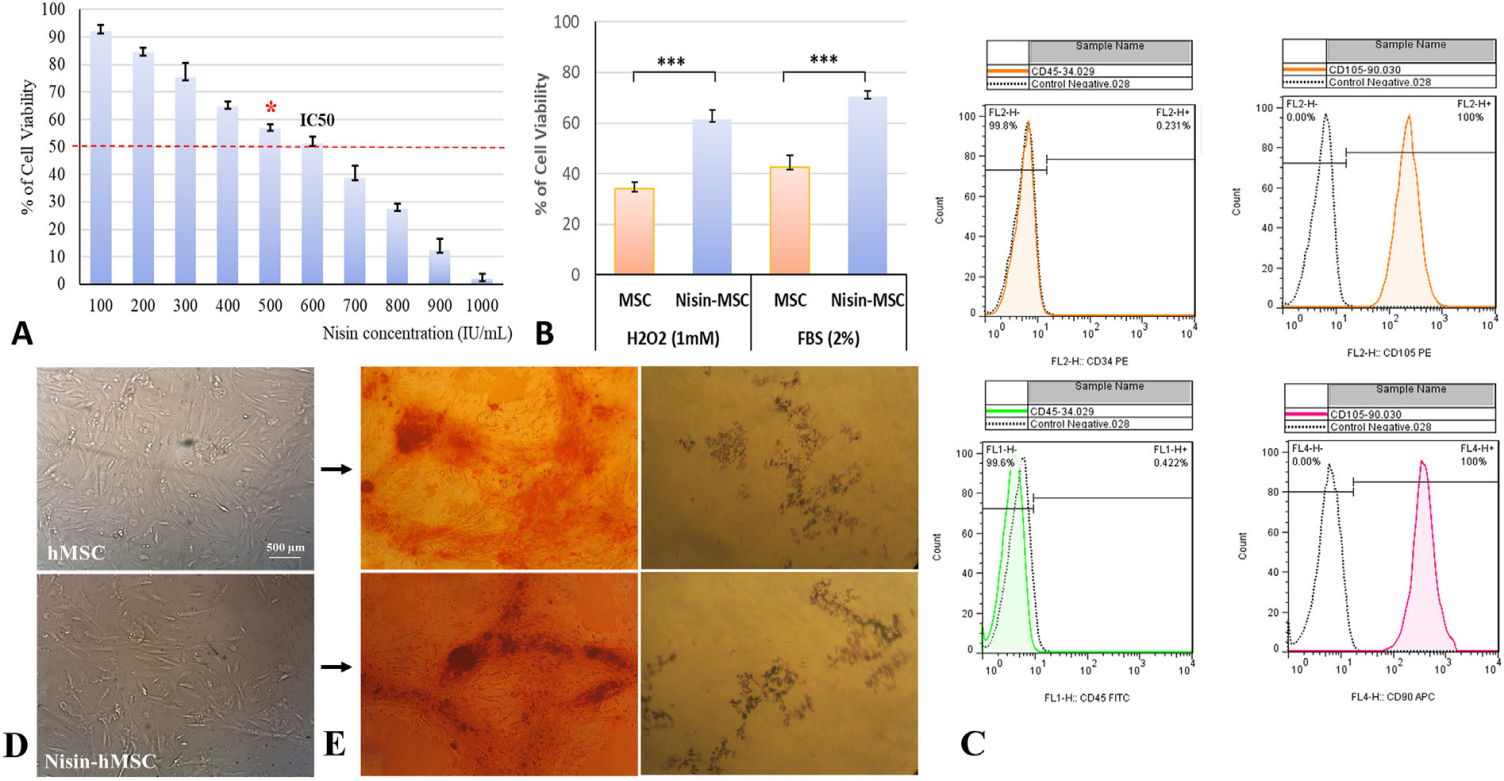
**MSCs Nisin preconditioning**. **A.** Investigating the survival of mesenchymal stem cells exposed to different doses of Nisin by MTT test. Treatment with concentrations of 100–1000 IU/mL of Nisin showed that the dose of 1000 IU/mL of Nisin was the lethal dose, the IC50 dose was 600 IU/mL, and the dose of 500 IU/mL was the optimal dose. **B**. Survival rates of MSCs and Pre-MSCs in the exposure to oxidative stress and serum deprivation. Comparing MSCs exposed to 1 mM H2O2 and 2% FBS for 24 h showed that MSCs preconditioned with Nisin became more stress-resistant. **C**. Immunophenotype of Nisin-hMSCs. CD markers profile of hMSCs after Nisin preconditioning was the same as before. Nisin-hMSCs were positive for CD29, CD44, and CD105, but negative for CD14, CD34, and CD45. **D.** Morphologic comparison of hMSCs and Nisin-hMSCs (10× magnification). After several passages and preconditioning, MSCs maintained their spindle and fibroblastic shape. **E.** Osteogenic and Adipogenic differentiation potential of hMSCs and Nisin-hMSCs. After several passages and preconditioning, MSCs maintained their osteogenic and adipogenic differentiation potentials.

A critical step in developing Nisin-preconditioned hMSC for therapeutic use is determining the maximal Nisin dose that maintains cell viability. We performed an MTT assay to evaluate the survival of hMSCs after exposure to varying concentrations of Nisin. The results revealed that the Nisin doses up to 500 IU/mL maintained over 90% MSC viability compared to untreated controls. However, viability declined in a dose-dependent manner, with only 2% survival at 1000 IU/mL, determined as a lethal dose. Nonlinear regression analysis provided a calculated IC_50_ value of 600 IU/mL for Nisin. Therefore, we identified 500 IU/mL as the sub-lethal/optimal Nisin preconditioning dose, as it potentiates antimicrobial function while preserving excellent MSC viability (Fig. 1 A).

##### 3.1.1.2 The effect of preconditioning on the protection of MSCs in stress conditions

Investigating the cytoprotective effects of Nisin preconditioning on hMSCs under cxidative stress and nutrient deprivation, MSCs experience harsh conditions like oxidative stress and nutrient deprivation upon transplantation. We hypothesized that preconditioning MSCs with Nisin may enhance their survival under challenging microenvironments.

MSCs underwent preconditioning with an optimal dose of 500 IU/mL Nisin for 24 h (Nisin-MSCs) or were untreated as control (MSCs). Both were subsequently subjected to oxidative stress through exposure to 1 mM H2O2 for 1 h. Additionally, MSCs were cultured in media with reduced serum (2% FBS) to model nutrient deprivation. Cell viability was quantified using the MTT assay post-treatment and expressed as a percentage relative to untreated control MSCs. Nisin-preconditioned MSCs exhibited significantly higher viability than control MSCs (85% vs 57%, p < 0.001) upon exposure to H2O2. Additionally, nutrient deprivation led to increased death of Control MSCs, while Nisin-MSCs maintained 84% viability (p < 0.001 vs. Control MSCs at 61% viability) (Fig. 1 B).

##### 3.1.1.3 Phenotypic Characterization of Nisin-hMSCs

To confirm there were no phenotypic changes following Nisin treatment, we analyzed the expression of standard MSC surface markers before and after preconditioning with 500 IU/mL Nisin using flow cytometry. Both untreated and Nisin-preconditioned MSCs showed positive expression (>90%) of CD29, CD44, and CD105, confirming their MSC identity. Additionally, both groups lacked expression (<2%) of hematopoietic markers CD14, CD34, and CD45. These results confirmed that the Nisin preconditioning does not alter the immunophenotype of MSCs (Fig. 1 C).

We also examined the morphology of hMSCs before and after Nisin treatment. Phase contrast microscopy showed that Nisin-hMSCs maintained the typical spindle-shaped, fibroblastic morphology identical to control MSCs even after multiple passages. This finding suggests that Nisin does not affect hMSC morphology or growth characteristics (Fig. 1 D).

##### 3.1.1.4 Multi-potency of Nisin-hMSCs

To evaluate whether Nisin preconditioning compromised the multipotent differentiation capacity of MSCs, we induced osteogenic and adipogenic differentiation in Nisin-preconditioned and non-preconditioned control hMSCs and examined lineage-specific staining. Both groups showed comparable staining for Alizarin Red (mineralization) and Oil Red O (lipid droplets) after differentiation, indicating preservation of osteogenic and adipogenic potential (Fig. 1 E).

In sum, viability, phenotypic, morphologic, and differentiation assessments confirm that preconditioning MSCs with 500 IU/mL Nisin primes their antimicrobial function without negatively impacting critical stem cell properties. These results support the therapeutic potential of Nisin-preconditioned hMSCs. Finally, Nisin preconditioning enhanced the survival of MSCs under oxidative stress and nutrient deprivation *in vitro*. This cytoprotective effect is likely mediated through increased metabolic activity and decreased apoptosis in Nisin-MSCs. These findings suggest that nisin preconditioning could improve MSC resistance to cytotoxic conditions and increase transplant retention and efficacy. Further, *in vivo* studies are warranted to confirm this potential translational benefit.

#### 3.1.2 Antibacterial activity of Nisin-MSCs and MSCs CMs

##### 3.1.2.1 Micro-broth dilution susceptibility assay

**Fig. 2:**
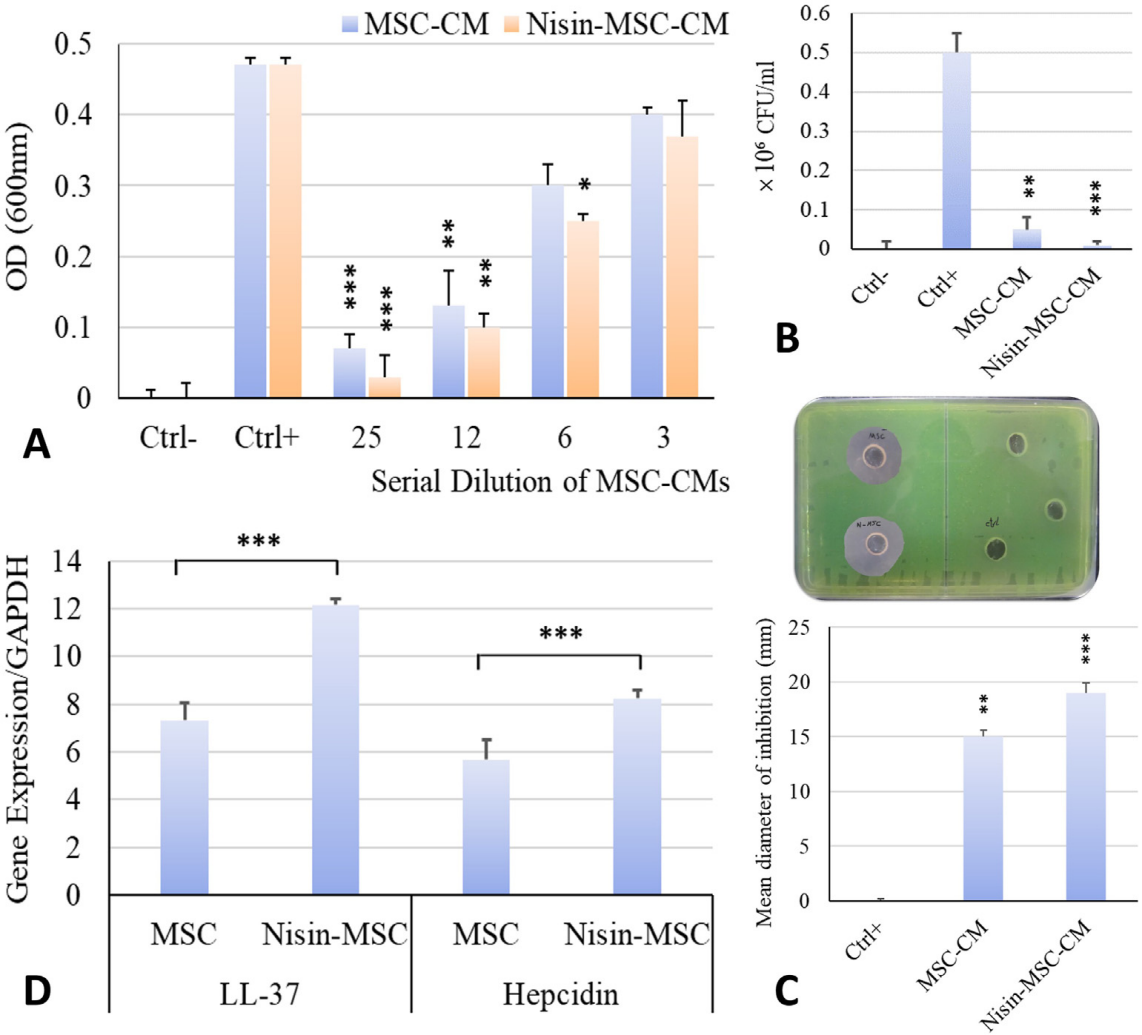
**Antibacterial activity of Nisin-MSCs and MSCs CMs. A.** Determining the minimum inhibitory concentration based on measuring the OD of *P. aeruginosa* culture treated with serial dilutions of CMs at 600 nm. The results were statistically significant compared to the control group (n = 3). **B.** The number of colonies of *P. aeruginosa* bacteria were cultured on blood agar plates with 10 μL of 25 times the concentration of each CM and then counted. **C.** The diameter of bacteria growth inhibition by MSC metabolites by well diffusion method in agar well. **D.** The relative expression of *LL-37* and *hepcidin* genes was determined using real-time PCR. The results were statistically significant compared to the control group (n = 3, ∗P < 0.05; ∗∗P < 0.01; ∗∗∗P < 0.001). Abbreviations: Ctrl; Control, MSC-CM; Metabolites of non-preconditioned MSC cells, Nisin-MSC-CM; Metabolites of MSC cells preconditioned with Nisin, antibiotics (Piperacillin/Tazobactam).

MSCs CMs were tested at varying dilutions for antibacterial effects against *P*. *aeruginosa*. The most concentrated/undiluted CM showed the most substantial inhibitory effect, significantly reducing Pseudomonas growth compared to controls (p < 0.001). At a 2-fold dilution, the CM exhibited significant but reduced antibacterial activity (p < 0.01). However, at a 4-fold dilution, only CM from Nisin-preconditioned MSCs (Nisin-MSC-CM) maintained significant antimicrobial effects (p < 0.05), while standard MSC CM lost efficacy. This concentration-dependent analysis demonstrates that factors secreted by MSCs inhibit *Pseudomonas* growth, with maximal effects in undiluted CM. Moreover, Nisin-pretreatment augmented the antibacterial potency of the MSC secretome, as Nisin-MSC-CM retained activity even with dilution (Fig. 2 A). Then, the number of colonies was counted from the mixture of bacteria treated with 15 μg of each CM on agar plates (Fig. 2 B).

##### 3.1.2.2 Well-diffusion method

The results showed both Nisin-MSC-CM and MSC-CM produced a clear zone of inhibition, indicating antibacterial effects. However, Nisin-MSC-CM generated a larger zone of inhibition compared to MSC-CM. The diameter of growth inhibition was ∼25 mm for Nisin-MSC-CM versus ∼15 mm for MSC-CM. So, preconditioning with Nisin causes the production of metabolites in the culture medium of MSCs, which increases the power of inhibiting the growth of *Pseudomonas* bacteria by at least 5 times (p < 0.001) (Fig. 2 C). This quantitative difference demonstrates Nisin-preconditioning enhanced the antibacterial properties of the MSC secretome. The larger zone of inhibition signifies greater diffusion of antibacterial factors secreted by Nisin-preconditioned MSCs.

##### 3.1.2.3 Antibacterial gene expression

The housekeeping gene *gapdh* showed no significant difference in expression between control and Nisin-preconditioned MSCs, so it was not affected by Nisin and served as internal control. The antimicrobial peptides *LL37* and *hepcidin* showed higher expression in Nisin-preconditioned MSCs compared to untreated controls. Specifically, there was a 3–5 fold upregulation of *LL37* expression and a 2–3 fold increase in *hepcidin* expression in the Nisin-preconditioned MSCs relative to controls. The relative fold-changes will be quantified using the ΔΔCt method comparing the Ct values of the target genes (*LL37*, *hepcidin*) to GAPDH between the two groups. Statistical analysis confirmed the significance of the observed differences in gene expression (p < 0.001). Hence, there was an evident upregulation of antimicrobial peptide expression, especially *LL37*, in Nisin-preconditioned MSCs compared to untreated controls (Fig. 2 D).

### 3.2 *In vivo* results

**Fig. 3:**
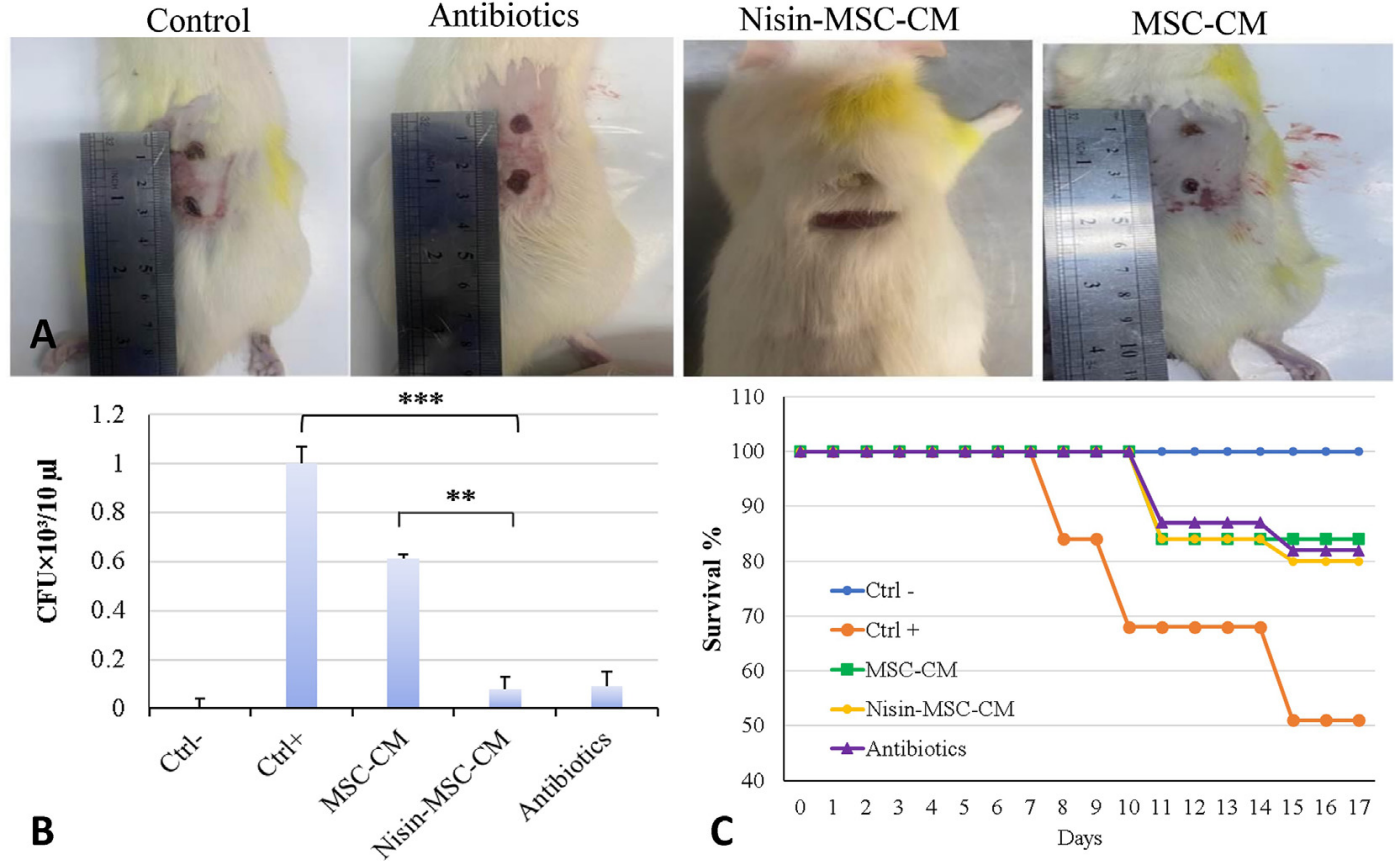
**Wound healing process. A**. Wound healing in treated rat groups, Control, antibiotics (Piperacillin/Tazobactam) receiving group, Nisin-MSC–CM receiving group, and MSC-CM receiving group, respectively. **B**. The effect of treatment with preconditioned CM on the inhibition of bacterial growth in rats. This shows that the wounded rats receiving Nisin-MSC-CM had significantly lower bacteria levels than those receiving MSC-CM (P < 0). (01) CM or PBS only (P < 0.001). The results were statistically significant compared to the control group. (n = 3, ∗P < 0.05; ∗∗P < 0.01; ∗∗∗P < 0.001). **C**. Preconditioning of MSCs with Nisin improves the survival rate of wound model rats. This shows that the wounded rats treated with Nisin-MSC-CM have a lower mortality rate than those treated with MSC or PBS alone. The survival rate of animals in each group was evaluated for 17 days, shown in the Kaplan-Meier diagram.

Four groups of rats were used to evaluate the antibacterial effects of MSC-CM against *P. aeruginosa* skin infections. In three treatment groups, two wounds were created on the back of each rat and subsequently infected with *P. aeruginosa* (Fig. 3A).

#### 3.2.1 Bacterial growth inhibition

Three days after infection, one group received topical application of unmodified MSC-CM mixed with a base ointment vehicle, another group received Nisin-MSC-CM, and the third opted for vehicle alone. Wound samples were collected and cultured on day 7 post-infection to quantify bacterial load and evaluate bacterial growth inhibition. Non-preconditioned MSC-CM significantly reduced bacterial burden (P < 0.001) compared to vehicle-treated rats, while Nisin-MSC-CM further decreased bacterial load (P < 0.001) (Fig. 3B). This suggests that Nisin preconditioning enhances MSC-CM's antibacterial properties against *P. aeruginosa* skin infections. The data provides evidence that factors secreted by Nisin-MSCs can effectively inhibit bacterial growth and promote bacterial clearance *in vivo*.

#### 3.2 2 Animal survival rate

Rat survival rate was assessed after 17 days, and wound healing rate was monitored every three days, and plotted on a Kaplan-Meier curve. Findings showed that rats treated with either MSC-CM formulation showed improved survival and faster wound closure than controls. The highest survival rate of 17 days was found in the group treated with Nisin-MSC-CM (∼80%), followed by MSC-CM (∼60%), while the positive control (∼50%) led to the survival of infected rats (Fig. 3C). Log-rank testing indicated the survival difference between Nisin-MSC-CM and other groups was statistically significant. This suggests that factors secreted by Nisin-preconditioned MSCs can effectively promote recovery and prevent mortality in this animal model of skin infection. Enhanced antibacterial properties of the Nisin-MSC secretome likely contributed to the survival benefit.

#### 3.2.3 CBC analysis during treatment

Following injection of cells into injured rats and controls, on the 5th day of CBC samples, some factors representing infection were evaluated based on the grouping.

There was no significant difference in the WBC level between the rats in the healthy control group and the two groups treated with concentrated Nisin-MSC-CM. In addition, the two groups were treated with the concentrated CM and the chemical test on day 5. But in the positive control group, WBC and CRP-q levels increased significantly, which indicated infection (Table 2).

**Table 2:** Biochemical analysis of animals after treatment.

Parameters	Experimental Groups					Normal Range
	MSC-CM	Nisin-MSC-CM	Antibiotics	Cont.+	Cont.-	
Hemoglobin	12.5 ± 2.6	13.1 ± 2.7	12.9 ± 0.9	13.4 ± 3.2	12.6 ± 4.1	11.5–16.1 g/dL
RBC	8.2 ± 0.6	8.2 ± 0.3	8.4 ± 0.2	8.3 ± 0.5	8.3 ± 0.09	6.76–9.75 × 10^6^/mm3
WBC	8.5 ± 0.9	8.2 ± 0.1	8.8 ± 0.09	14.3 ± 0.7	8.9 ± 0.4	6.6–12.6 × 10^3^/mm3
Patelets	245 ± 11	274 ± 12	221 ± 8	245 ± 11	238 ± 14	150–460 × 10^3^/mL
Hematocrit	43.4 ± 4.5	41.2 ± 8.2	44.1 ± 7.2	43.3 ± 6.8	41.3 ± 3.9	35.2–49.8 %
CRP-q	2 ± 0.05	3 ± 0.03	3 ± 0.07	9 ± 0.01	2 ± 0.08	≤5

RBC, Red blood cell count; WBC, White blood cell count; CRP, C-reactive protein, MSC, Mesenchymal stem cell; CM, Conditioned medium; Cont.+, Positive Control (Ciprofloxacin); Cont.-, Negative Control; Antibiotics, Piperacillin/Tazobactam.

An analysis of blood samples from rats treated with MSC-CMs (MSC-CM or Nisin-MSC-CM), an Antibiotics, or a control treatment revealed several markers of infection and inflammation. The positive control group had a high WBC count (14.3 × 10^3^/mm^3^) indicating infection. Nisin-MSC-CM and MSC-CM groups showed normal WBC levels (8.2–8.8 × 10^3^/mm^3^), suggesting antimicrobial effects controlled infection. CRP levels, serving as an indicator of infection, exhibited a notable increase in the positive control group (9 mg/L) compared to the normal (<5 mg/L). The treatment groups exhibited mild CRP elevations (2–3 mg/L), indicating less severe infection. Hemoglobin, hematocrit, RBC, and platelet counts were within normal ranges for all groups, meaning the absence of notable impacts on platelet count or coagulation. Overall, the results demonstrate that both MSC-CM treatments, especially Nisin-MSC-CM, suppressed infection markers compared to the untreated positive control. These results align with the *in vivo* data showing enhanced antimicrobial effects of the conditioned media treatments. The differences between Nisin-MSC-CM and MSC-CM effects were minor based on this analysis.

## 4 Discussion

However, using MSCs in clinical applications comes with challenges, one of which is their short life span in harsh environment of inflammation [11]. To address this issue, researchers have explored the use of Nisin as a preconditioning agent for MSCs before transplantation. Nisin is a bacteriocin with broad-spectrum antimicrobial activity, making MSCs more resistant and longitude their life span [7,10,12]. In this study, we aimed to investigate the antibacterial effects of Nisin-preconditioned MSCs against common bacterial contaminants. By utilizing the antimicrobial properties of Nisin, the safety and efficacy of MSC-based therapies were sought to enhance by minimizing bacterial infection risks during transplantation. Proposed mechanisms of Nisin's antimicrobial activity include inhibition of pore formation in bacterial membranes and cell wall synthesis [13].

It has been established through studies that MSCs' preconditioning with low levels of H2O2 can augment their therapeutic efficacy in promoting wound healing. MSCs preconditioning with 50 μM H2O2 for 12 h optimally enhances viability, migration, and wound healing capacity compared to untreated BMSCs. Preconditioning activates adaptive mechanisms in MSCs that make them more resistant to the harsh conditions of injured tissues. It allows them to survive better, migrate, and stimulate regeneration when transplanted into wounds [14]. Finding the optimum dose of a treatment agent is crucial. Our results showed that treatment with concentrations of 100–1000 IU/mL of Nisin affected the survival of the cells. The Nisin's lethal dose was 1000 IU/mL, the IC50 dose was 600 IU/mL, and the sub-lethal/optimal dose was 500 IU/mL. A vital strength of the study was the systematic optimization of the Nisin preconditioning protocol, carefully titrating the dose to identify 500 IU/mL as optimal for potentiating antimicrobial function while maintaining excellent hMSC viability and multipotency. Thorough characterization confirmed that Nisin-preconditioned hMSCs keep their phenotype, morphology, and differentiation capacity, supporting their translational potential. These results align with previous studies showing that Nisin doses of 0.5–1 μg/mL (equal to 500–1000 IU/mL) can increase *LL37* and *defensin* expression in MSCs by 3–6 fold [15].

Recent studies have shown that Nisin treatment enhances the therapeutic activity of MSCs through cytokine modulation and direct cytotoxic effects on cancer cells. However, the direct effects of MSCs in Nisin preconditioning are not well defined. Researchers were preconditioned human bone marrow-derived MSCs with Nisin and induced *S. aureus* infection [6,16]. This study explored an innovative approach of using the antimicrobial peptide Nisin to precondition hMSCs and enhance their therapeutic potential against *P. aeruginosa* infections. The results demonstrate that Nisin preconditioning primes the antibacterial properties of MSCs without impacting their viability or stem cell characteristics. We hypothesized that the Nisin prerequisite would enhance the native antibactericidal capacity of MSCs against common bacterial contaminants, without affecting their viability and multipotent differentiation potential. Clarification of this may contribute to the development of new Nisin-based precursor strategies to reduce the risk of bacterial contamination of MSC therapies. The immunophenotype of Nisin-hMSCs was found to be the same as before, with the cells being positive for CD29, CD44, and CD105, but negative for CD14, CD34, and CD45. The morphologic comparison of hMSCs and Nisin-hMSCs showed that after several passages and preconditioning, MSCs maintained their spindle and fibroblastic shape. The osteogenic and adipogenic differentiation potential of hMSCs and Nisin-hMSCs was also maintained after several passages and preconditioning. These results suggest that Nisin treatment does not affect the morphology, immunophenotype, or differentiation potential of MSCs.

A study revealed that BMSCs preconditioning with δ-tocotrienol (δ-TT), a vitamin E isoform, enhances the therapeutic potential of wound healing by modulating specific molecular pathways by stimulating cell proliferation, angiogenesis, and migration [17,18]. Probiotic metabolites and MSCs have been shown individually to promote wound healing processes such as improved wound closure and tissue regeneration. However, combining probiotic metabolite preconditioning of MSCs may have synergistic effects and improve therapeutic outcomes beyond either treatment alone. The enhanced viability of MSCs preconditioned with probiotic metabolites like Nisin suggests that the metabolites may augment MSCs' survival and potency [15]. The results of this study will provide crucial insights into the effects of Nisin preconditioning on the inherent antibacterial properties of MSCs and their lifespan. Understanding the mechanisms by which Nisin potentiates the antibacterial effects of MSCs could lead to improved cell preparation protocols and reduced bacterial contamination risk for stem cell therapies.

Studies have documented that human BM-MSCs and ASCs exhibit antibacterial effects, indicating their potential as adjunctive therapies for infections like septic arthritis and osteomyelitis. Some natural metabolites can further enhance the innate antimicrobial capacity and therapeutic value of BM-MSCs and ASCs against bacterial pathogens such as *Staphylococcus aureus* [[18], [19], [20]]. Here, bacterial growth inhibition results demonstrated that topical application of Nisin-MSC-CM to infected wounds significantly reduced bacterial load compared to MSC-CM or PBS, highlighting the potent antibacterial effects. Statistical analysis reveals that the differences between treatment and control groups were significant. CMs in the most concentrated state had the most remarkable effect on the growth of *Pseudomonas* bacteria and inhibited it significantly (p < 0.001). Inhibition of bacterial growth in half of the initial concentration of CMs was also significant (p < 0.01). While only Nisin-MSC-CM at a concentration of one quarter significantly increased bacterial growth to restrain (p < 0.05).

A study investigated that the hUCMSC CM mitigated the development of imipenem resistance in *P. aeruginosa*, which highlights the potential of MSC therapies to prevent antibiotic resistance emergence. Inhibition zone testing showed imipenem was far more effective against bacteria in hUCMSC CM (20 mm zone) than control/LL-37 media (12 mm zone) [[21], [22], [23]]. Here, the agar diffusion assay results provide visual and quantitative evidence that CM from Nisin-preconditioned MSCs has superior antibacterial activity against *P. aeruginosa* compared to CM from untreated MSCs. The diameter of growth inhibition was ∼25 mm for Nisin-MSC-CM versus ∼15 mm for MSC-CM. Therefore, preconditioning with Nisin stimulates metabolite production by MSCs, which increases the power of *Pseudomonas* growth inhibition by at least 5 times (p < 0.001). This consequence further supports the bacterial growth inhibitory effects of factors secreted by Nisin-preconditioned MSCs.

MSCs express and secrete various antimicrobial peptides (AMPs), including *LL-37*, *hepcidin*, *human beta defensin-2* (*hBD-2*), and *lipocalin 2* (*Lcn2*), which exert antibacterial effects. The production and release of these AMPs indicate that they may play a crucial role in MSC's innate antimicrobial capacity. However, the regulation and relative contributions of specific MSC-derived AMPs to direct bacterial killing require further investigation [[24], [25], [26]]. The *in vitro* assays showed upregulation of antimicrobial peptide expression (*LL-37* and *hepcidin*) in Nisin-preconditioned MSCs. More importantly, the conditioned medium from Nisin-preconditioned hMSCs exhibited enhanced antibacterial effects against *P. aeruginosa* versus non-preconditioned hMSCs, evidenced by larger inhibition zones in diffusion assays and reduced bacterial load. These results mechanistically demonstrate that Nisin preconditioning augments the innate antimicrobial capacity of the hMSC secretome.

Most research investigations have documented that chemokines and growth factors released from MSCs, such as TGFβ and VEGF, impact wound healing [[27], [28], [29]]. The *in vivo* findings in the rat wound infection model further substantiate the therapeutic promise of this approach. The macroscopic wound healing progression in the different treatment groups showed that the Nisin-MSC-CM group shows faster wound closure and healing than other groups. The antibacterial effects of topical application of CMs were quantified by measuring bacterial burden in wound cultures. Nisin-MSC-CM decreased bacterial levels significantly compared to non-preconditioned MSC-CM or control, aligning with enhanced antimicrobial activity. The survival curves over 17 days were compared for the different groups. An explicit separation shows improved survival with Nisin-MSC-CM treatment compared to other groups. The macroscopic, microbial burden and survival data are consistent and complement each other, demonstrating superior wound healing and antibacterial effects with Nisin-MSC-CM versus standard MSC treatments. Overall, *in vivo* findings provide compelling evidence that Nisin preconditioning enhances the therapeutic potential of the MSC secretome to improve wound healing outcomes in the context of bacterial infections.

Commonly, a bacterial infection can be diagnosed, prognosed, and monitored using CRP and WBC, with CRP being more sensitive [[30], [31], [32], [33]]. Our CBC test analysis demonstrated that elevated WBC count and CRP in the untreated positive control group indicate active bacterial infection. In contrast, the MSC-CM treatment groups maintained WBC and CRP levels close to the normal range and the negative control. This suggests that the antimicrobial factors secreted by MSCs help control infection systemically and limit inflammatory markers. The comparable results between standard MSC-CM and Nisin-preconditioned MSC-CM align with the wound culture findings, showing both inhibit bacterial growth, but Nisin may confer a modest enhancement. Normal red blood cell and platelet values imply that the MSC-CM treatments do not adversely affect other hematological parameters.

Some limitations should be acknowledged. The study focused solely on augmenting antibacterial effects against *P. aeruginosa*. Testing against other prominent nosocomial pathogens like *S. aureus* could further generalize the findings. Additionally, only a short time *in vivo* treatment was evaluated, while a thorough assessment of wound healing over longer durations could better characterize therapeutic efficacy. Nevertheless, this pioneering work provides a strong foundation for developing Nisin-preconditioned hMSC therapies to combat antibiotic-resistant infections.

## 5 Conclusion

In conclusion, this study highlights a novel preconditioning strategy to improve the antimicrobial activity of hMSCs while preserving their regenerative capacity. Focusing on synergies between stem cell therapies and antimicrobial peptides holds tremendous promise for managing clinical challenges at their interface. The multifaceted therapeutic effects of Nisin-preconditioned hMSCs could provide innovative solutions to tackle nosocomial infections and promote tissue repair, meriting further research and development.

## Funding

This study was not funded by any grant from an institute.

## Authors’ contribution

Conceptualization: Halabian and Aghamollaei; Methodology: Enayati, Saeedi; Data curation: Halabian and Saffarian; Data interpretation: Halabian, Saeedi, Aghamollaei, and Saffarian; Resources: Halabian, Enayati and Aghamollaei; Supervision: Halabian; Project administration: Halabian, Saeedi and Enayati; All authors reviewed and approved the manuscript.

## Data availability

The datasets used and (or) analyzed during the current study are available from the corresponding author upon reasonable request.

## Declaration of competing interest

The authors declared no conflicts of interest.
